# Supplementary material for: Capturing the Liquid-Crystalline Phase Transformation: Implications for Protein Targeting to Sterol Ester-Rich Lipid Droplets
Source: Membranes (Basel). 2022 Sep 28;12(10):949. doi: 10.3390/membranes12100949 (PMC9607156; doi:10.3390/membranes12100949)
Supplement: Supplementary file 1 [file membranes-12-00949-s001.zip › membranes-1922730-supplementary.pdf]

Supplementary Material

Capturing the liquid-crystalline phase transformation: Implications for protein targeting to sterol ester-rich lipid droplets

R. Jay Braun[1], Jessica M.J. Swanson[1]*

1. Department of Chemistry, University of Utah, Salt Lake City, Utah 84112-0850, USA *Corresponding author: j.swanson@utah.edu

a) b)

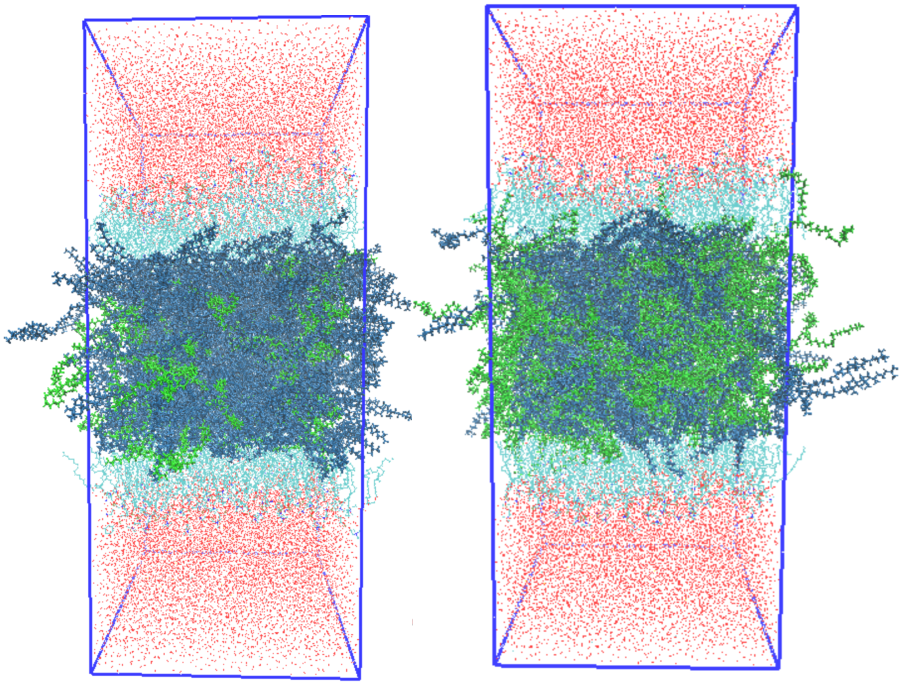

Figure S1. (a) 90:10 CHYO:TG system, and (b), the 50:50 CHYO:TG system during the 1st μs. CHYO is blue, TG is green. Trilayer systems are used to represent LD's, see *Methods* section for more details.

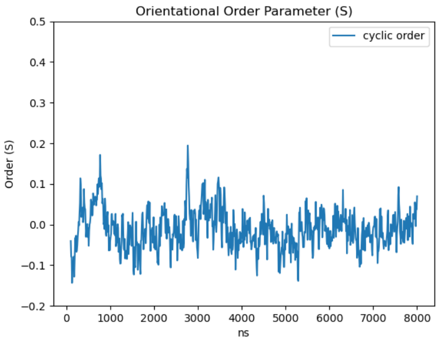

Figure S2. The orientational order parameter (S) for the 50:50 CHYO:TG LD averages 0, indicating no ordering has occurred.

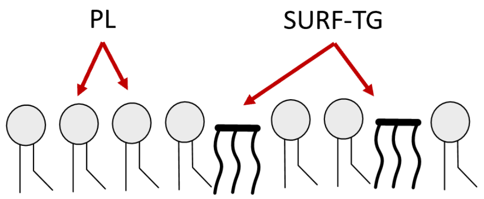

Figure S3. Representation of SURF-TGs in the PL monolayer. The SURF-TGs behave as membrane components

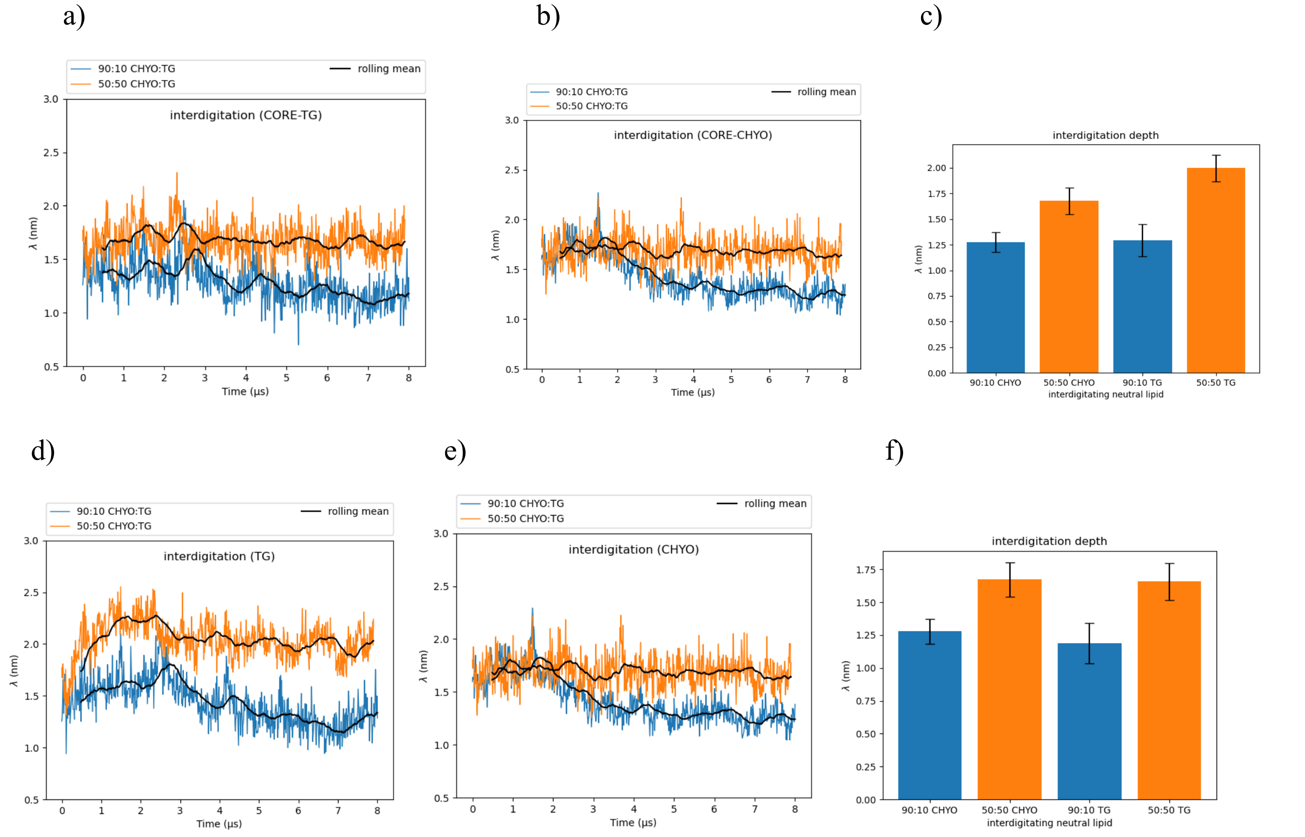

Figure S4. (a) Interdigitation as a function of time for CORE-TG, and (b) CORE-CHYO. (c) Interdigitation depth as a function of time for total-TG, and (d) Average interdigitation after initial equilibration of 3 μs to allow for CHYO ordering to occur. (d) Interdigitation total-TG and (e) total-CHYO. (f) Average CORE interdigitation depth after initial equilibration of 3 μs.

SI table S1. The average magnitude of interdigitation of neutral lipids for multiple LD species. The calculation were taken after 4 μs to allow for equilibration

**LD type**	**90:10 CHYO:TG LD**	**50:50 CHYO:TG LD**	**Pure TG LD**
SURF-TG	0.130±0.014	0.605±0.039	~1.27
CORE-TG	1.188±0.155	1.657±0.139	~1.83
TG total	1.294±0.157	1.998±0.0.127	~2.42
CHYO	1.278±0.097	1.678±0.129	

Standard errors SURF: *90:10 TG: 0.001, 50:50 TG: 0.001*

Standard errors CORE: *90:10 CHYO: 0.005, 50:50 CHYO: 0.008, 90:10 TG: 0.008, 50:50 TG: 0.007*

Standard errors total: *90:10 CHYO: 0.005, 50:50 CHYO: 0.008, 90:10 TG: 0.008, 50:50 TG: 0.007*

a) b)

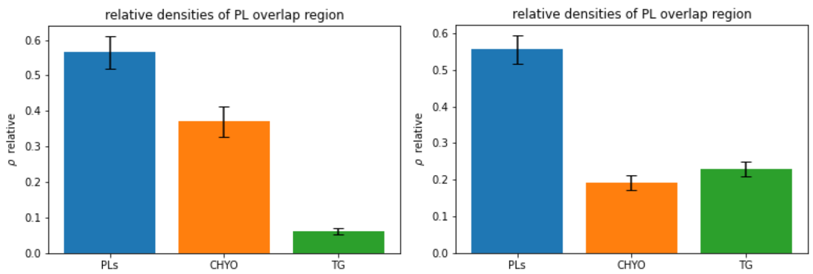

Figure S5. The interdigitation of the neutral lipids and phospholipids. (a) ρ_{rel} for the 90:10 system and (b) ρ_{rel} for the 50:50 system.

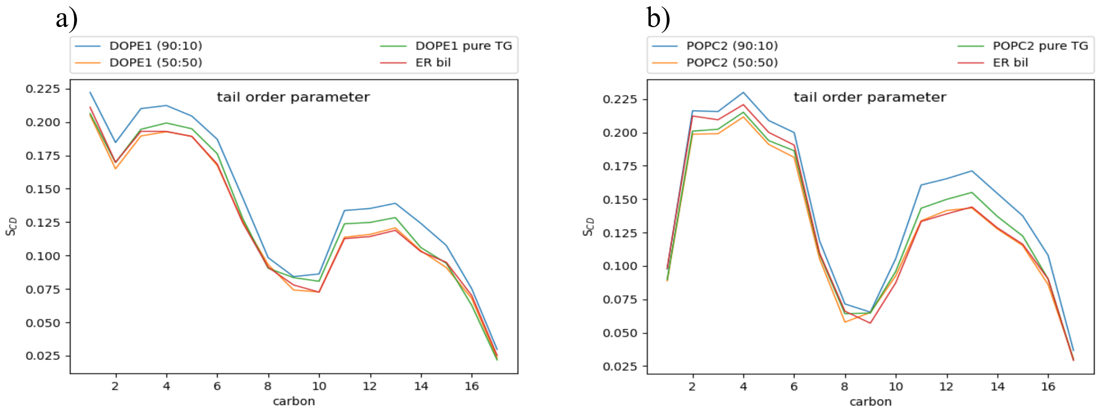

Figure S6. PL tail order parameters for (a) DOPE and (b) POPC. In these plots, we included the order parameters of and ER bilayer (red) and a pure-TG LD (green)

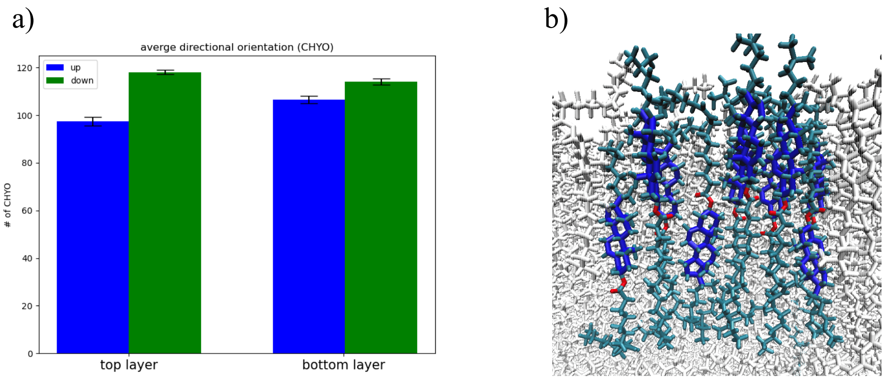

Figure S7. Calculation of the orientation of CHYO molecules per smectic layer (a), and a screenshot of CHYO in one of the layers displaying the alternating orientation (b).
